# Five Cases of Ununited Fracture, in Which Excision of the Ends of the Bone, or the Seton, Was Employed
*American Journal of Medical Science, Nov. 1835.


**Published:** 1836-10-01

**Authors:** 


### ON UNUNITED FRACTURES.

### Five Cases of Ununited Fracture, in which Excision of the Ends of the Bone, or the Seton, was employed.*

These cases are of sufficient interest to deserve a particular notice at our hands. They are contained in the American Journal of the Medical Sci-

* American Journal of Medical Science, Nov. 1835.

ences, in which they have been reported by an intelligent physician, Dr. Kirkbride. It is gratifying to observe the manner in which America is contributing her share to the stores of facts, which, rightly appreciated, form the foundation of all that is positive and valuable in medicine. Not a Number of this Journal is now published, in which American contributions to its pages are not found, and they are daily becoming more important.

We shall first present a concise account of the cases, and then offer what remarks we have to make upon them.

CASE I. *Ununited Fracture of the Femur near its middle—Excision of the Ends of the Bone—Cure.*

John M'C. æt. 26, admitted February 30, 1833. He had been in the country five years, and had suffered at first from intermittent fever. In 1828 he had two or three of his ribs broken, and in 1830 an arm, all of which united in the usual period.

On the 6th of July, 1832, he was crushed by the falling of a bank of earth, six and a half feet high, which fractured both thighs, near their middle—the right leg just below the knee—and the right patella. There was in addition, an extensive wound of the perineum. A bandage was applied to each limb; splints not more than a foot long to each thigh, and the injured leg merely kept at rest upon pillows. The limbs were examined at the end of three weeks, and the same dressings re-applied and suffered to remain till the 1st of November. When removed, the left femur was found to have united with great deformity, the ends of the bone overlapping, and the limb shortened two inches—the patella and the tibia were also firm, but no union in the right femur.*

On his admission, the right limb was one inch shorter than the left—the fragments of bone were moveable, and were not in contact, a large portion of callus had been thrown out about the seat of injury, and appeared to prevent their approximation, and rendered the deformity very striking. His general health was pretty good.

On the 23rd of March the operation of removing the ends of bone was performed by Dr. T. Harris. It was found necessary to remove a large portion of new bone, that had been deposited in the part, and which added materially to the difficulty of the operation; this done—the end of each fragment was excised by means of the chain saw, and the two ends were then readily brought into apposition—the edges of the wound were approximated,

* This case is a fair illustration of the effects of treatment without splints, for here the short splints, applied *only* to the thigh, can hardly be considered as treatment *with* splints. How any body laying claim to common sense, and possessed of the smallest modicum of experience, can seriously countenance Mr. Radley's "anti-splint" absurdities, is more than we can understand. If an animal's thigh is fractured and Nature allowed to do her best, a pretty job she makes of it. Most frequently she unites the bone, but how? The broken ends overlap in an extreme degree, and such distortion ensues as splints are both intended and able to prevent. Persons must have a strange idea of the action of muscles who can believe that when left to themselves they will not draw the portions of the broken bone from the right line that the whole bone describes.

the limb placed in Hartshorne's modification of Boyer's splints for fracture of the femur, and the parts covered with a poultice. This was employed for three months, after which a pasteboard splint was made use of. The wound healed by the 1st of November. Early in December there was an appreciable stiffening in the limb; soon after he was directed to leave his bed, and commenced walking with crutches, the thigh being protected by the splint. On the 1st of May, 1834, he was sent into the country. In September he called at the hospital. The limb felt perfectly firm, but was less strong than the other. He used a cane in walking, and a cork sole one inch high made the length of the two limbs correspond. During the progress of the cure the patient had eight or nine attacks of erysipelas.

Case II. *Ununited Fracture of both Bones of the Fore-arm—Excision of the ends of the Bones, with partial success—Subsequent Formation of the same state of Parts—Application of the Seton—Perfect Recovery.*

Solomon M'D. æt. 24, admitted April 14, 1833. In August, 1832, both fore-arms were fractured by a water-wheel in two or three places; in the left fore-arm the middle fracture was a compound one, the bones projecting for more than an inch through his coat. The right arm was retained in splints during two months; union took place, and it gradually regained its strength. Splints were never applied to the left, but it was kept at rest upon a pillow for nearly six months, while various measures were resorted to, to remove the inflammation, &c.

On his admission, the arm was useless; the line of direction, both of the radius and ulna, was so much altered, and the fragments had passed each other so far, that neither friction nor the seton could be resorted to. The operation of Mr. White was, therefore, performed by Dr. T. Harris on the 24th of April; the ends of both bones were turned out and excised, so that upon replacing them, they were accurately in apposition. The callus thrown out between the bones, and in some measure uniting them, rendered the operation rather tedious and painful.

The arm was kept in a curved splint, but occasionally, from attacks of erysipelas, &c., it was necessary to remove all dressings and merely keep the limb quiet on a pillow. In the latter end of September the ulna had united perfectly, and the ends of the radius were in contact. But on the 13th of November he insisted on being discharged.

After leaving the house, he was employed as a drover, still keeping on the splints which he had used in the hospital. Early in July, 1834, he fell from a fence, breaking up the newly-formed callus, and suffered much pain for some time afterwards. On the 29th of October, he was again admitted into the hospital, the portions of the radius and ulna perfectly moveable, and appearing to have their ends rounded off, from the motion to which they had been subjected. As the fragments now corresponded with each other, it was determined to employ the seton, and accordingly a portion of a skein of silk was introduced between each of the ununited fractures by Dr. Harris, on the 2d of November.

In December, there was some stiffening of the radius; none of the ulna. In January there was evident stiffening of the latter.

The progress of the case was interrupted by cough, diarrhœa, and hæmoptysis; the consequence was that, at the end of February, the radius was less firm than it had been a fortnight previously.

Although he had another attack of hæmoptysis, the bones now became more firm, and on the 27th of May, when he was sent into the country, both bones were united, and he had ceased for some days to wear splints of any kind. The last seton was removed nearly three weeks before he was discharged.

CASE III. *Ununited Fracture of the Humerus near its Neck—Cure by the Seton.*

John E. æt. 24, admitted May 10, 1834. On the 25th of February, he was thrown down from a rail-road car. His right humerus was fractured obliquely, just below the head of the bone, and both bones of the forearm, midway between the wrist and elbow; splints were placed upon the arm, and it was secured to the body by means of a roller; the forearm he carried in a box padded with cotton. In two months he was able to use the forearm. The splints were kept upon the arm for six months; they were then removed, as the fracture was supposed to have united. He was never able to use it, however, and in January, 1834, met with a person, who, discovering that he had ununited fracture, proposed the use of a seton, which was accordingly tried, but finding no union at the end of eight weeks it was removed. Nothing further was done till he entered the hospital.

From an examination of the cicatrices upon the arm, it was evident that the seton had been merely passed under the integuments, and at least an inch below the edges of the separated bone. The ends of bone were easily moveable, and appeared to have a ligamentous connexion.

The operation was performed by Dr. T. Harris on the 17th of May. After making an incision down to the part, he used a strong-pointed steel instrument, with a firm handle, for perforating the ligament, and then passed a portion of a skein of silk between the fragments, by means of a long seton-needle.

On the 18th some oozing of blood.

On the 23rd, erysipelas.

On the 25th, bleeding from a vessel deep in the wound.

On the 4th of June, the erysipelas had disappeared.

On the 28th, some stiffening at the seat of fracture—four firm splints round the part.

On the 3rd of August, the seton was removed. On the 15th of September, the splints were also taken away. The bone then appeared perfectly firm.

On the 29th of October, he was discharged. The arm was strong.

CASE IV. *Ununited Fracture of the Bones of the Leg, removal of a portion of the shaft of the Fibula, and excision of the points of the other fragments—Cure.*

Felix L. æt. 30, admitted Aug. 1, 1834, with ununited fracture of the bones of the leg. On the 24th of July, 1833, he had received a compound fracture of both bones of the left leg, with protrusion of one of the fragments of the tibia. The limb was kept in splints for five months. Extensive ulceration took place,—portions of the tibia exfoliated, but no union occurred. The limb was shortened, and at times painful—the ulceration had healed—but he was only able to walk with crutches.

On dissection it was found that the fracture of the tibia had occurred two inches and a half above its lower point—that the fracture had been oblique, and that a considerable portion of bone had exfoliated—that the fracture in the fibula was also very oblique, and just above that part which enters into the composition of the joint; after the accident the upper fragment of fibula had slipped down upon the outer side of the lower, so as to resemble on a superficial inspection, the external malleolus; and in this situation a firm ligamentous union had taken place before the process of exfoliation was completed in the tibia, the fragments of which were thus kept so widely separated, that nature was unable to repair the injury by a deposite of osseous matter.

The application of the caustic potass on the nearest point of the tibia was ineffectually tried. There was no alternative but to excise a sufficient portion of the fibula to allow the approximation of the separated fragments.

The operation was performed on the 25th of November, and the exposure of the parts confirmed exactly the views which had been previously taken. The tibia was first exposed, and the projecting points of each fragment taken off, so that the opposite surfaces might come fairly into contact. This part of the operation, showed the precise extent of fibula that must be removed to permit this approximation. The ligament connecting the two portions of fibula was next separated, and after removing the point of the lower fragment, the upper was turned out, and one and a half inches taken from its inferior extremity. The different portions of both bones then came accurately into contact with each other.

On the 8th of January, there appeared to be some stiffening of the fibula, and a few spiculæ of bone had been discharged from between the ends of the tibia.

24th. Erysipelas of the leg.

February 6. The fibula had united—no union in the tibia—the ulcerated surfaces just healing. Pasteboard splints.

In the latter part of February, a shoe, with a heel sufficiently high to make the length of the two limbs correspond, was procured for him; the limb was then enclosed in moistened firm pasteboard, which hardening, formed a complete case for the parts, and he was directed to walk and bear as much weight as possible upon the diseased limb, with a view of hastening the deposite of callus.

On the 11th of April, he went into the country, the union of the tibia not being yet firm. The pasteboard splint had been removed. After several weeks he returned. He could walk without assistance. The stiffness of the ankle-joint and the depression of the great toe, consequences of the original injury and of the treatment necessary for it, were the greatest annoyance. The shortening of the limb was remedied by a very high cork sole.

Case V. *Ununited Fracture of the Femur—Excision of the Ends of Bones—Death from Purulent Deposits in the Lung.*

Benjamin W., æt. 24, admitted Feb. 14th, 1835, with ununited fracture of the os femoris, three inches below the trochanter minor. The bone was broken on the 23d of August, 1832, by the falling of a bank of earth upon him. He had previously suffered from intermittent fever.

For the fracture, long splints were first applied, and removed at the end of six weeks, as union was supposed to be perfect. He commenced walking with crutches, the toes only of the injured limb reaching the ground, and he soon became sensible that the fracture had not united. Violent extension was afterwards made on two or three different occasions, and the limb confined in splints, or some other apparatus for a great many weeks, but all ineffectually. His health suffered at first from the confinement, but he gradually regained his strength.

On the 4th of March, the operation of excising the ends of bone was performed by Dr. Hewson. The operation was necessarily tedious and difficult.

On the evening of the 15th he had a rigor, followed by fever and sweating. After this he had at intervals other rigors—became jaundiced—had cough, with dyspnœa—low fever—and died on the 20th.

*Dissection.* The two portions of the femur were in apposition; the cup-like cavity that was forming as a substitute for the joint, was about two and a half inches in diameter, and half an inch deep, irregular at its margins, covered with cartilage, and was three inches below the upper part of the trochanter major. Along the lower fragment an abscess extended to within two and a half inches of the knee; in nearly the whole of this extent the bone was denuded of periosteum, rough, and partially surrounded by pus. Three small pieces of detached bone were discovered.

There was no phlebitis found. Sero-purulent fluid in the left pleural cavity, with some yellow lymph on the pulmonary pleura. Puriform fluid in the right pleural cavity, and many purulent deposits in both lungs.

The preceding cases are interesting as a collection of facts. The main points which such cases are required to determine are two:—the causes of the want of union; and the mode of remedying it.

The causes of non-union are clearly of two kinds:—those which relate to the state of the constitution and system at large—and those which relate to the limb.

1. The constitutional causes which tend to prevent proper union in bones, are debility, no matter how induced, and the existence of some other action in the system, which seems to supersede the reparation of bone.

*a.* The operation of constitutional debility is very well exhibited in the following instances, both of which fell under our own observation.

*Case* 1. A man received a simple fracture of both bones of one leg. At the end of six weeks there was only soft union. We ascertained that he had previously been a great porter drinker. We immediately gave him a large allowance of porter, (he had previously had an ordinary quantity,) and in a very short time the union was perfect.

*Case* 2. A woman met with a simple fracture of both bones of one leg. She was fat, rather plethoric, and accustomed to good living. She had spasms of the limb after the accident. Her surgeon bled her freely for these. The spasms ceased—she became at once much weakened—and the bones never united.

We introduce these cases as an illustration of the principle we have laid down. Other illustrations will readily occur to the surgeon of much reading or of much experience. The subject of the fifth case in the present report had intermittent fever prior to his meeting with the fractured thigh, and that is the only ostensible cause for its non-union. Some persons appear, independently of obvious constitutional weakness, to repair fractures of bones with difficulty. It is singular that those who are peculiarly prone to fracture do not seem to exhibit more impediments than usual to its reparation.

*b*. The effect of some other action going on in the system has been frequently noticed in the case of pregnancy. Many instances of non-union in pregnant women have been placed on record. We have witnessed two.

Obvious constitutional debility—peculiarity of constitutional state—and the existence of some other action of importance in the economy, appear, then, to be the chief general causes of the want of ossific union after fracture.

2. The local impediments to that restorative process seem to be tolerably well established.

*a*. If the ends of bone are constantly disturbed, the process of consolidation is interfered with, and a false joint is the consequence. It has been found, by experiment, that if, after fracturing the thigh-bone of a rabbit, the ends of bone be rubbed on each other every day for a certain time, ossific union does not take place. Many cases of the same description have been related. It may be concluded from these numerous and positive facts, that rest is an essential element in the production of bony union.

*b*. Position is another element. A knowledge of the process of reunion informs us, that the more nearly the ends of bone are in perfect apposition, the more readily are both the external and internal callus produced. In the four-footed mammalia, reunion occurs with comparative facility, and extensive malposition scarcely seems to interrupt the process. We have made many experiments this last Summer on the reparation of broken bone. We have chiefly selected the femur of the rabbit as the subject of our observations, and in the museum of the School of St. George's Hospital, in Kinnerton Street, is a series of preparations displaying the phenomena that take place. No precautions were taken to prevent distortion, and the action of the adductor muscles has in all cases drawn the upper fragment greatly upwards and inwards. Yet the junction of the bones has occurred in all but one instance where a false joint formed. The preparations display distinctly the difficulty experienced in forming a good external callus, when the portions of bone overlap each other much. If, in addition to this overlapping, they are also separated by a material interval, the difficulty in question must be proportionally augmented.

It is probable that mal-position was the cause of the non-union in the first, second, and fourth, of the cases in the Pennsylvania hospital.

Want of rest and improper position are the main local causes of defective osseous union, at least in simple fractures. In compound fractures, the disturbing circumstances are more numerous; and we may observe that the reparation of compound fractures has not been sufficiently investigated.

The examination of the causes of non-union naturally leads us to the consideration of the best mode of preventing it. If general debility and the absence of sufficient rest and of proper position are the principal of those causes, the judicious invigoration of the system, and the maintenance of rest and of due position are the obvious measures of prevention. On the former we need not dwell. The successful application of the latter depends on the use of appropriate apparatus.

It is not our present object to review the various local modes of treating fractures. That would be out of place in a paper which is devoted to a short exposition of principles. But an accurate acquaintance with the anatomical changes that occur in the natural process of reparation is indispensable, to enable us to select an apparatus with propriety, and to apply it with precision. Too frequently, the surgeon merely does what he has seen others do before him, and can make no pretensions to a philosophical knowledge of the laws of growth he is assisting, or impeding.

1. The first stage of the reparation of fracture is the effusion of blood, and of its components, into the contiguous parts. The ends of bone lie amidst torn tissues and extravasated blood.

2. A certain degree of inflammatory action begins to be set up, and the tissues injected with blood, and consolidated by effusion, hem in the ends of bone, which have made for themselves a cavity. The fracture may thus be said to be included in a cyst.*

3. The periosteum of the respective portions of bone, near their fractured ends and the cellular tissue, and the contiguous muscular tissue, become agglutinated together, and of a sort of ligamentous consistence. Into this strong capsule (for a capsule it resembles) the parietes of the cyst have been converted, and it holds the bones together. The cavity is now more or less diminished by the thickening and insensible contraction of this capsule.

If a false joint forms, the process of reparation appears to be nearly arrested at this point. The capsule, indeed, becomes thicker and stronger, and bony matter is even deposited in parts of its substance. But the cavity in which the bones lie continues, and the ends of the bone become gradually rounded off by friction, and either undergo a sort of ivory transformation, or are tipped with a strong fibrous membrane.†

4. The thickening of the capsule goes on, and gradually the whole of its cavity is superseded by a fibro-cartilaginous deposit. This adheres to a deposition in the medullary cavity, at the extremity of each fractured portion. The old term callus is now applicable to the uniting material. It surrounds the ends of the bones, is inseparably blended with the vascular periosteum and adherent muscle, and forms, in the language of Dupuytren and Breschet, a clasp which binds the bones together. But it also extends between the bones, supposing (as almost always happens) that an interval is left, and adhering, sooner or later, to a deposition contained in their medullary cavities, it tends still further to unite them.

During this time, osseous matter has been deposited in the substance of the capsule, and, indeed, of the "callus." Slowly the whole is converted into bone, and then begins the modelling process of absorption, reducing the unseemly mass of new bone, new fashioning the exterior, and re-establishing a continuous internal cavity. We say little upon these points, because they are unconnected with our present subject.

It is clear, from a review of the changes which occur in the reparation of bone, that any apparatus intended to promote them, should secure rest to

---

* This is very distinct in a preparation in the School of Kinnerton Street.

† There is a preparation in Kinnerton Street, exhibiting the arrest of reparation at this point, and a consequent false joint. The rabbit died on the 19th day.

the limb and apposition to the fragments, without the employment of undue pressure. If rest and apposition are secured, it is difficult to conceive what useful purpose can be served by pressure to any amount. Yet we constantly see limbs jammed and squeezed in splints as though they were in vices, and the tighter the surgeon draws the bandages, the more certain he feels, or seems to feel, that union will occur. This is a great error. The periosteum, the neighbouring tissues of all kinds, have to take on a great increase of vascularity and action. If the broken limb of an animal is injected with size and vermilion, the vessels are seen ramifying in profusion, the old arteries being increased in dimensions, and new vessels carrying red blood being developed. The process of reparation is very analogous to that of growth. What should we think of the man, who swathed in unyielding cinctures the growing cylindrical bone of a young creature, and told us it was with the view of promoting its ossification? Yet this would not be one whit more absurd than the practice constantly adopted in fractures. The mistake has evidently sprung from an imperfect knowledge of the natural mode of reparation, and from practically confounding the benefits derived from promoting apposition of the fragments, with the effects of pressure beyond that limit.

In short, if an apparatus secures comparative immobility to the bones, and keeps them pretty closely together, it does all that an apparatus can do or ought to do. When it does more, it interferes with Nature, and we may be certain that she knows her business better than a cobbling surgeon can teach it her.

But a false joint has formed, or the bones have been united by the fibro-cartilaginous substance, into which sufficient osseous matter has not been secreted. What is to be done?

The plans that have been tried may be resolved into two:—

Pressure and apposition, which keep the bones together, and secure them in the most favourable situation for union.

And the induction of more vascular action in the capsule or the callus.

Theoretically speaking, pressure is most adapted for those cases in which perfect apposition has not previously been obtained. So far as we have seen, it has succeeded best in the thigh and arm, where powerful muscles interfere with the rest and apposition of the portions of the broken bone. In some cases of non-union, the ends of bone are either actually separated, or admit of being separated to some distance from each other. It is obvious that the chances of union would be increased by whatever brought them together, and kept them so. We have not yet sufficient experience of cases of non-union, to enable us to determine precisely what are the cases adapted for pressure. But of this we may be sure. As it is the mildest and the safest mode of treatment, it should always be first resorted to. If it does not cure, still, by diminishing the space between the bones, it lightens the work for the seton or excision.

The induction of increased vascular action in the callus and the surrounding parts, has been attempted in various ways, and with various success. Rubbing the ends of the bone together—cutting down on them, and irritating them by caustic—excising them—passing a seton between them, have been tried with greater or with less success. The seton and excision have been found the most powerful, and are now generally adopted.

It would be difficult to determine the respective merits of these two methods. The seton is only adapted for those cases in which the capsule has little bone, and in which the callus, such as it is, admits of being easily perforated. Where there is excessive malposition, the seton must be useless. Excision only promises in a case of this description to bring the bones in apposition. Excision, on the other hand, is difficult and dangerous where the bone is situated deeply amidst muscles, as the humerus and femur are. On the whole, the seton is the easier and the milder plan of treatment, and experience has proved it to be far from unsuccessful.

In the second and third of the cases before us, the seton effected a cure—in the second, after excision had failed. The former case was one of fracture of both bones of the fore-arm—the latter, of fracture of the humerus near its neck.

In the first and the fourth cases, excision was performed with perfect success. The former was one of fracture of the femur—the latter of both bones of the leg.

In the fifth case, excision of portions of the femur proved fatal. The result can hardly be considered accidental, because it is likely to follow such an operation, and must be calculated on in estimating its disadvantages.

The following is a summary of the cases and the results.

*Case* 1. Fracture of the Femur—Excision—Reported cure after eighteen months—necessary to use a cork sole one inch high.

*Case* 4. Fracture of both bones of the leg—Excision—Reported cure (not very positive) after nine months—necessary to use " a very high" cork sole.

*Case* 5. Fracture of the Femur—Excision—Death from Purulent Deposits on the sixteenth day.

*Case* 2. Fracture of both bones of the Fore-arm—Seton—Cure after six months.

*Case* 3. Fracture of the Humerus—Seton—Cure after four months.

One point only remains. Dr. Kirkbride observes:

" Erysipelas has been an attendant on every case of operation for ununited fracture that we have seen, with one exception, and that one survived only a short period. The same fact is noticed in many of the reports of similar cases."

We have read these cases with interest, and presented them to our readers with pleasure. If any derive an useful hint from the observations we have made on them, our object will be amply served. We are anxious to diffuse amongst the mass of the profession exact notions, and philosophical habits of thought. This is a high aim, if we do not succeed in effecting it. We leave to others the taste for the marvellous and the obscure.

